# Impact of ethanol on odor detection thresholds relevant to No- and Low-Alcohol (NoLo) wines: a combined sensory and computational approach in simplified static hydroalcoholic model wine

**DOI:** 10.1016/j.crfs.2026.101563

**Published:** 2026-09-12

**Authors:** Laura Brian, Anthony Santacreu, Come de Tournadre, Michel Malcor de Pierrefeu, Jeanne Laronche, Jean-Stéphane Condoret, Audrey Devatine, Séverine Camy, Olivier Geffroy

**Affiliations:** a Univ Toulouse, Ecole d’Ingénieurs de Purpan, PPGV, Toulouse, France; b Univ Toulouse, Toulouse INP, CNRS, LGC, Toulouse, France

**Keywords:** Rotundone, Isoamyl acetate, 2-phenylethanol, Volatility enhancement factor, NRTL activity coefficient model

## Abstract

The effect of ethanol on odor detection thresholds (ODTs) in fully and partially dealcoholized (NoLo) wines remains largely unexplored. This study used a combined sensory and thermodynamic approach in simplified static hydroalcoholic wine model systems at 0, 6, and 12 % v/v ethanol with three aroma compounds. Orthonasal ODTs were measured using a 3-AFC ascending method. NRTL-based thermodynamic modeling was used to derive a volatility enhancement factor (VEF), defined as the volatility ratio between ethanol levels, and to estimate headspace concentrations at ODT. ODTs were significantly increased at 6 and 12 % v/v for all compounds, with no difference between these levels. Relative to 0 % v/v, ODT fold increases ranged from 5.7 to 6.1 for rotundone, 4.2-5.9 for isoamyl acetate, and 9.8-10.8 for 2-phenylethanol. VEFs were compound-dependent, being highest for rotundone (VEF_12_ _→_ _0_ = 86.7), intermediate for isoamyl acetate (5.4), and lowest for 2-phenylethanol (3.4). ODT increases were mainly thermodynamically driven for rotundone, whereas perceptual and ethanol masking effects likely dominated for 2-phenylethanol, with isoamyl acetate intermediate. These results indicate that key aroma compounds remain perceptually relevant in NoLo wines despite substantial volatile losses. More broadly, this approach provides a framework for studying aroma behavior in static hydroalcoholic systems.

## Highlights

• Ethanol significantly increased ODTs for three key wine aroma compounds.
• NRTL modeling enabled the introduction of a volatility enhancement factor (VEF_a_._→0_)
• Changes in ODT for rotundone mainly driven by thermodynamic effects.
• For 2-phenylethanol, perceptual masking effects prevailed.
• High values of VEF_a_._→0_ may compensate aroma losses due to dealcoholization process

## Graphical abstract

**Figure:**
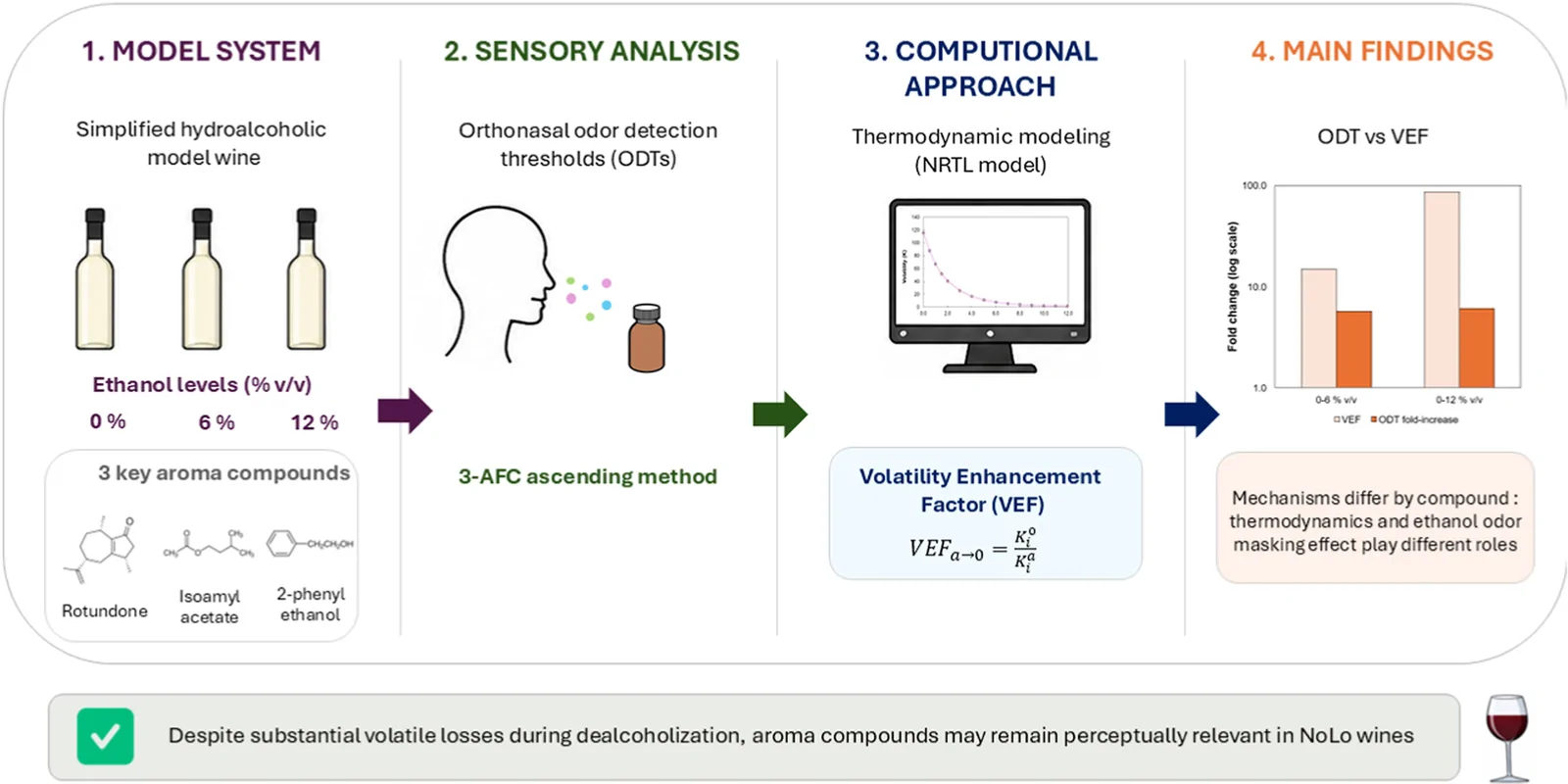

## 1 Introduction

In the current context of declining wine consumption and structural overproduction, the wine industry is exploring diversification strategies to attract new consumers (Del Rey and Loose, 2023). Driven by the success of alcohol-free beers (Anderson, 2023), increasing attention is being paid to totally dealcoholized (no) and partially dealcoholized (low) wines, commonly referred to as NoLo wines (Shaw et al., 2023).

Only a limited number of physical techniques are currently available for wine dealcoholization, including membrane-based processes and vacuum distillation methods (Akhtar et al., 2025). Among membrane-based approaches mostly used for partial dealcoholization (El Rayess and Mietton-Peuchot, 2016), reverse osmosis allows ethanol and water to permeate through a semi-permeable membrane while retaining most wine constituents (*e.g.*, aroma compounds and phenolics). Despite its misleading terminology, osmotic distillation involves the transfer of volatile compounds, including ethanol, in the vapor phase across a hydrophobic membrane driven by a vapor pressure gradient. Pervaporation and diafiltration are additional processes, the former involving selective permeation followed by evaporation under reduced pressure, and the latter consisting of pressure-driven filtration with continuous solvent addition to enhance the removal of low-molecular-weight compounds such as ethanol. Regarding distillation-based methods, which are generally implemented to achieve total dealcoholization, simple vacuum distillation has been used for decades. However, it results in substantial losses of aroma compounds (up to 90 %) due to its low selectivity between ethanol and other volatile constituents (Kumar et al., 2025). To limit this loss, distillation using Spinning Cone Column (SCC) or Gas-Only Low-Pressure (GOLO) are gaining in popularity within the industry (Alises et al., 2025). In these processes, multi-stage or fractionated stripping under reduced pressure enables the partial recovery and reintroduction of volatile compounds, and thus improves overall product quality. Recently, supercritical CO_2_ has been proposed as a promising approach for separating ethanol from other volatile compounds in distillates, due to its high ability to recover aroma (Geffroy et al., 2020).

The removal of ethanol is known to significantly affect wine sensory properties, leading to reduced viscosity, body and fullness, and to an increased perception of tannin harshness (Jordão et al., 2015; Longo et al., 2017; Schmitt and Christmann, 2022). Depending on the process applied, ethanol extraction may account for up to 20 % of the initial wine volume and can simultaneously result in the concentration of non-volatile constituents such as polyphenols and organic acids (Belisario-Sanchez et al., 2009). Nevertheless, wines with reduced alcohol levels (up to 2.8 % and 7.0 % v/v for Chardonnay and Syrah, respectively) are generally not rejected by consumers in comparison with standard wines (Geffroy et al., 2022). To rebalance in-mouth sensations, sweetening typically through sucrose addition up to 40 g/L is the most commonly implemented and effective strategy to achieve consumer acceptance (Podworny et al., 2024).

To produce fully dealcoholized wines of acceptable quality through distillation when aroma recovery is not feasible, exogenous flavorings are often added, resulting in products labelled as “beverages made from dealcoholized wine”. Nevertheless, the effect of ethanol on aroma detection and perception in the NoLo alcohol concentration range has not been studied to date. The only available study on Malbec wines, well above this ethanol range, showed that increasing alcohol content from 10.0 to 12.0 to 14.5-17.2 % v/v differentially affects aroma composition and sensory perception, with most attributes decreasing while some increase (Goldner et al., 2009). As summarized by Ickes and Cadwallader (2017), ethanol concentration significantly alters the molecular structure of water-ethanol matrices and strongly influences aroma release and partitioning, generally decreasing volatile aroma concentration in the headspace in static systems likely to reflect the so-called “first nose” conditions in wine tasting (*i.e.*, aroma perception without agitation). On the opposite, according to the same authors, the headspace concentration in aroma compounds is increased in dynamic conditions that approximate “second nose” conditions (*i.e.*, aroma perception with agitation). This work also highlighted that odor detection thresholds (ODTs) for a given aroma compound generally increase with ethanol concentration, from water to 10 %, 23 % and 40 % v/v. However, direct comparisons remain challenging, as these thresholds were generally determined across distinct studies using different sensory panels.

In addition to altering the volatility of aroma compounds, ethanol also has a detectable odor that can influence overall aroma perception. This perceptual contribution may interact with other odorants, potentially masking or modulating their perception (Ferreira et al., 2021).

Ethanol also acts directly at the receptor level by modulating olfactory receptor cell function. This pharmacodynamic dimension has been characterized for several compounds in model spirit solutions containing 20 and 40 % v/v ethanol (Wang and Cadwallader, 2024). These studies were conducted in dynamic systems using gas chromatography (GC) combined with mass spectrometry (MS) and olfactometry (O), and panels composed of 3 to 4 trained judges.

To our knowledge, no work has been undertaken in the NoLo ethanol range under static conditions. Direct analysis of aroma release in the headspace under hydroalcoholic conditions remains methodologically demanding, requiring complex experimental setups (Ettre, 2001). A thermodynamic approach-based equilibrium calculations with Non-Random Two-Liquid (NRTL) model calculations could provide a simpler way to estimate aroma partitioning between liquid and gaseous phases in static mode as a function of ethanol concentration (Renon and Prausnitz, 1968). Such a model is an activity coefficient model widely used in process engineering for representing non-ideal liquid-phase behavior via local composition concepts. Combined with sensory experiments, this approach could help to decipher the respective contributions of thermodynamic and perceptual effects in static experimental design for which no direct assessment of pharmacodynamic effects is possible.

The aim of this work was to implement such approaches in simplified hydroalcoholic model wine systems at three ethanol concentrations (0, 6, and 12 % v/v), using three key wine aroma compounds (*i.e.*, rotundone, isoamyl acetate, and 2-phenylethanol).

## 2 Materials and methods

### 2.1 Chemicals, selection of aroma compounds and sample preparation

Ultra-pure water from a Milli-Q system (Millipore, Burlington, Massachusetts, USA) was used in the study. Absolute ethanol (≥99.8 %) was provided by VWR International (Rosny-sous-Bois, France). Rotundone (≥99 %) was supplied by Firmenich (Geneva, Switzerland) while isoamyl acetate (≥98 %) and 2-phenyl-ethanol (≥99 %) were purchased from Sigma-Aldrich (Saint Quentin Fallavier, France). These three compounds were selected as key wine aroma compounds to cover a diversity of origins (grape-derived or produced during fermentation), a wide range of ODTs spanning from ng/L to mg/L, and distinct chemical and aroma families with rotundone, isoamyl acetate and 2-phenylethanol kwown to impart pepper, banana and rose-like (dried rose) notes, respectively (Cordente et al., 2018; Plata et al., 2003; Wood et al., 2008). Another advantage of selecting rotundone is that specific anosmia has been reported for this compound with a clear bimodal distribution of ODT in a recent study which is not the case for the two other molecules (Denat et al., 2025).

The test solutions were prepared in Milli-Q water and absolute ethanol at three concentration levels (0, 6 and 12 % v/v), corresponding to fully dealcoholized, partially dealcoholized and conventional wines, respectively. Stock solutions of the aroma compounds were prepared in absolute ethanol at compound-specific concentrations to limit the increase in ethanol content of the spiked samples to a maximum of 0.5 % v/v. Blank samples were not solvent-matched for this addition, in agreement with the ASTM E679 method (ASTM, 2004), which defines blanks as samples containing the media without the stimulus, without prescribing carrier-solvent matching.

Samples were prepared at least 12 h before testing to reach equilibrium and stored at 4 °C in the dark until used. Prior to sensory evaluation, samples were removed from refrigerated storage and allowed to equilibrate for 2 h at the testing temperature (20 °C), matching the temperature used for thermodynamic modeling. A volume of 10 mL of each test solution was placed in a 30 mL brown glass flask with a plastic screw cap supplied by Embelia (Charenton-le-Pont, France).

A posteriori GC-MS and GC-FID analyses using previously proposed methods (Mattivi et al., 2011; Ortega et al., 2001) suggested limited compound-dependent deviations from target concentrations.

### 2.2 Panelists and procedure for ODT determinations

The effect of ethanol on ODTs was assessed over three consecutive sessions (one per aroma compound) conducted over a two-week period in Toulouse, southwest France, using panels mainly composed of young adults. For rotundone, the panel included 31 participants (21.2 ± 2.3 years, mean ± SD), comprising 45 % women and 55 % men. For isoamyl acetate, 55 panelists participated (22.3 ± 3.1 years, mean ± SD), with 64 % women and 36 % men. Finally, the panel for 2-phenylethanol consisted of 57 participants (22.3 ± 4.7 years, mean ± SD), including 51 % women and 49 % men. A small number of participants took part in more than one session. The number of participants meets usual recommendations (ASTM, 2004), providing sufficient coverage of inter-individual variability for reliable threshold determination.

No financial compensation was provided. Ethical approval was not required for this type of sensory study at the École d’Ingénieurs de Purpan. However, all participants were informed about General Data Protection Regulation (GDPR) requirements and provided written consent.

Orthonasal ODTs for the three compounds were determined using a three-alternative forced-choice (3-AFC) ascending method (from lowest to highest concentration), following ASTM E679 (ASTM, 2004) standard. This method was chosen as it provides individual ODTs in a single session and supports modelling of ethanol effects with replicate data.

Concentrations ranged from 1.9 to 2899.2 ng/L for rotundone, 0.51 to 781.25 μg/L for isoamyl acetate, and 0.38 to 585.94 mg/L for 2-phenylethanol, with a geometric progression factor of 2.5 between successive levels, which represents 9 series of samples for each compound. These concentration ranges were defined based on preliminary trials conducted in water with four trained assessors and previously reported ODT values for these compounds (Denat et al., 2025; Tempere et al., 2011; Tsukatani et al., 2003). Although ethanol is expected to influence ODTs, the same test solution concentrations were used across all ethanol levels to ensure consistency and comparability.

ODT measurements were conducted under controlled conditions (20 °C, neutral sensory rooms with white walls and natural light). Panelists were seated sufficiently apart to prevent interaction and samples were coded with random three-digit numbers. Each session was divided into three subsessions, one for each ethanol concentration. During a session, participants evaluated nine series of samples across all ethanol levels, with the order of subsessions randomized for each panelist. A 5-min break was imposed between subsessions.

Panelists were instructed to smell the samples in the prescribed order, without agitation to ensure static conditions, and then identify the spiked sample. There was space for comments on the reason for their choice. For each participant and subsession, the occurrence of each descriptor (banana-like, rose-like, pepper-like and ethanol-like) was recorded after lemmatization and triangulation (Abric, 2005) based on whether it was cited at least once across the nine series. It was hypothesized that this metric could reflect the physiological masking effect of ethanol, with descriptors associated with the target compounds being cited less often relative to ethanol-like descriptors as ethanol concentration increases.

Given the static system and panel-based 3-AFC tests, pharmacodynamic effects of ethanol at the receptor level were not accessible, and observed changes in ODT reflect primarily thermodynamic and perceptual interactions. Indeed, such effects are likely to be minimal due to the absence of continuous odorant flux and the fact that concentrations were not renewed in the headspace.

### 2.3 Computational approach

Calculations were based on thermodynamic phase equilibrium (liquid-vapor equilibrium (LVE)) at fixed liquid composition, temperature and pressure conditions, governed by the equality of chemical potentials of each component (equation (1)).

(1) $$\mu_{i}^{V}\left(T,P,\bar{y}\right)=\mu_{i}^{L}\left(T,P,\bar{x}\right)\;i=1,\ldots ,n$$

Equality (1) can be reformulated in terms of equality of fugacity (equations (2) and (3)).

(2) $$f_{i}^{V}\left(T,P,\bar{y}\right)=f_{i}^{L}\left(T,P,\bar{x}\right)\;i=1,\ldots ,n$$

(3) $$y_{i}P\Phi_{i}^{V}\left(T,P,\bar{y}\right)=x_{i}\gamma_{i}{\left(T,\bar{x}\right)P}_{i}^{sat}\left(T\right)\;i=1,\ldots ,n$$

Where x_i_ = molar fraction of component i in the liquid phase, y_i_ = molar fraction of component i in the vapor phase, $P_{i}^{sat}$ = saturated vapor pressure of component i at T, T = temperature, P = pressure and $\gamma_{i}$ and $\Phi_{i}^{V}$ = activity coefficient and fugacity coefficient of compound i respectively, measuring deviations from ideality in liquid and vapor phase, respectively.

In the present case, at low pressure, the gas phase behaves as a perfect gas, thus $\Phi_{i}^{V}$ is equal to 1. Solving this system of equations is enabled through the use of the activity coefficient model (NRTL). This model describes non-ideal mixtures by accounting for intermolecular interactions (equation (4)). The key intermediate parameter *G*_*ij*_ *and τ*_*ij*_ were used to compute the activity coefficient (equations (5) and (6)).

(4) $$\mathrm{Ln}\;\gamma_{i}=\frac{\sum_{j=1}^{n}\tau_{ji}G_{ji}x_{j}}{\sum_{k=1}^{n}G_{ki}x_{k}}+\sum_{j=1}^{n}\frac{x_{j}G_{ji}}{\sum_{k=1}^{n}G_{kj}x_{k}}\;\left(\tau_{ij}-\frac{\sum_{k=1}^{n}x_{k}\tau_{kj}G_{kj}}{\sum_{k=1}^{n}G_{kj}x_{k}}\right)\;i=1,\ldots ,n\;;\;j=1,\ldots ,n$$

(5) $$\tau_{ij}=\frac{C_{ij}^{0}+C_{ij}^{T}\left(T-273.15\right)\;}{RT}\;i=1,\ldots ,n\;;\;j=1,\ldots ,n$$

(6) $$G_{ij}=\exp\left(-\alpha_{ij}\times \tau_{ij}\right)\;i=1,\ldots ,n\;;\;j=1,\ldots ,n$$

α_ij_ corresponds to the non-randomness parameter of the mixture, set to 0.3 according to previously published recommendations (Renon and Prausnitz, 1968) for associated polar mixtures. Binary interaction parameters C_ij_ describe the interactions between molecules i and j, and were determined from experimental data reported in the literature (Puentes et al., 2018) or estimated using predictive models (Table 1). These parameters may depend on temperature or not, (C^T^ = 0 in this latter case).

**Table 1:** NRTL binary interaction parameters used for liquid-vapor equilibrium calculations. For rotundone and isoamyl acetate, values were estimated using predictive model (UNIFAC modified) while for 2-phenylethanol, experimental data were used (Puentes Mancipe, 2017). $C_{ij}^{T}$ and $C_{ji}^{T}$ were set to 0 for 2-phenylethanol–solvent binary systems due to insufficient experimental data over a reliable temperature range.

Binary system		$C_{ij}^{0}$ (cal/mol)	$C_{ji}^{0}$ (cal/mol)	$C_{ij}^{T}$ (cal/mol)	$C_{ji}^{T}$ (cal/mol)
i	j				
Ethanol	Water	34.02	850.12	-1.80	5.65
Rotundone	Water	2580.41	6629.32	-6.49	13.00
Rotundone	Ethanol	470.99	1285.16	-9.30	2.00
Isoamyl acetate	Water	1490.25	3999.34	-7.21	8.32
Isoamylacetate	Ethanol	325.76	560.18	-5.47	2.79
2-phenylethanol	Water	−1363.50	5732.50	0	0
2-phenylethanol	Ethanol	−1747.30	3693.20	0	0

The reliability of NRTL parameters for aroma compounds highly diluted in hydroalcoholic mixtures has been established in prior experimental and modeling work. Puentes et al. (2018) reviewed and modeled the vapor-liquid equilibrium of numerous aroma compounds diluted in ethanol-water mixtures across the full miscibility range, and Zanghelini et al. (2022) further validated this approach through dedicated experimental measurements and NRTL-based modeling of a terpene alcohol diluted in hydroalcoholic matrix. These studies support the applicability of the NRTL model and its associated parameters to the highly diluted hydroalcoholic model wine considered in the present work.

The limited availability of experimental data in the literature resulted in an insufficient temperature range for reliable estimation of the C^T^ parameters for 2-phenylethanol-solvent binary system. Therefore, it was reduced to 0.

Equilibrium calculations were carried out using the *Simulis Thermodynamics®* calculator software (Baudouin et al., 2008). The thermodynamic model was used to determine the vapor-phase composition at specified temperature, pressure, and liquid-phase composition. It enabled calculations of partition coefficient of compounds (K, equivalent to volatility) between liquid and vapor phase for each value of the alcohol composition as follows (equation (7)).

(7) $$K_{i}=\frac{y_{i}}{x_{i}}$$

For each studied compound, the volatility was assessed at 0.0, 0.5, 1.0, 1.5, 2.0, 3.0, 4.0, 5.0, 6.0, 7.0, 8.0, 9.0, 10.0, 11.0, and 12.0 % v/v ethanol fraction. To quantify the thermodynamic enhancement in aroma volatility induced by ethanol reduction, a volatility enhancement factor (VEF) was defined (equation (8)).

(8) $${VEF}_{a\to 0}=\frac{K_{i}^{0}}{K_{i}^{a}}$$

where $K_{i}^{a}$ and $K_{i}^{0}$ are the volatility of aroma i at ethanol concentrations a % and 0 % (v/v), respectively. In this work, a = 6 or 12 % v/v. A value of VEF > 1 indicates enhanced volatility at 0 % v/v ethanol content compared to a % v/v ethanol content.

The concentration of molecules in the vapor phase in equilibrium with a liquid concentration equal to the ODT was determined as an indicator of intrinsic olfactory potency, reflecting the number of molecules required in the vapor phase to trigger detection independently of liquid-phase concentration. For this, the ideal gas law was used with the molar fraction in the vapor phase at the mixture bubble point at ambient temperature (20 °C), equation (9).

(9) $$C_{i}=\frac{y_{i}\times P_{bubble}}{RT}$$

where P_bubble_ is the bubble pressure of the mixture at fixed temperature.

### 2.4 Statistical treatments

Given the probabilistic nature of odor detection, individual thresholds were estimated using an approach derived from the ASTM E679 BET method. For each participant, the thresholds were calculated as the geometric mean between the lowest concentration at which three consecutive correct responses were obtained and the immediately preceding concentration (Wise et al., 2008; Denat et al., 2025). In addition, no more than one incorrect response was allowed at higher concentrations (ASTM, 2004; Wise et al., 2008; Denat et al., 2025). Panelists failing at the highest concentration were assumed to succeed at 2.5 times that level, whereas those succeeding at the lowest concentration were assumed to fail at 2.5 times lower concentration. For each aroma compound and ethanol concentation, the panel threshold was estimated as the geometric mean of the individual thresholds.

Individual thresholds were log-transformed, and linear mixed models (LMMs) were used to assess the effect of ethanol as a fixed factor for each compound. On the same data, Hartigan's test was conducted at 0 % v/v ethanol to assess the hypothesis of unimodality (Hartigan and Hartigan, 1985). The citation frequencies of descriptors were analysed using a Chi-square test followed by a Marascuilo post-hoc procedure. Data analyses were performed using XLSTAT 3.1 software (Addinsoft, Paris) except for Hartigan's test that was carried out using the diptest package in R.

## 3 Results

### 3.1 ODT of the three compounds at 0 % v/v

**Fig. 1:**
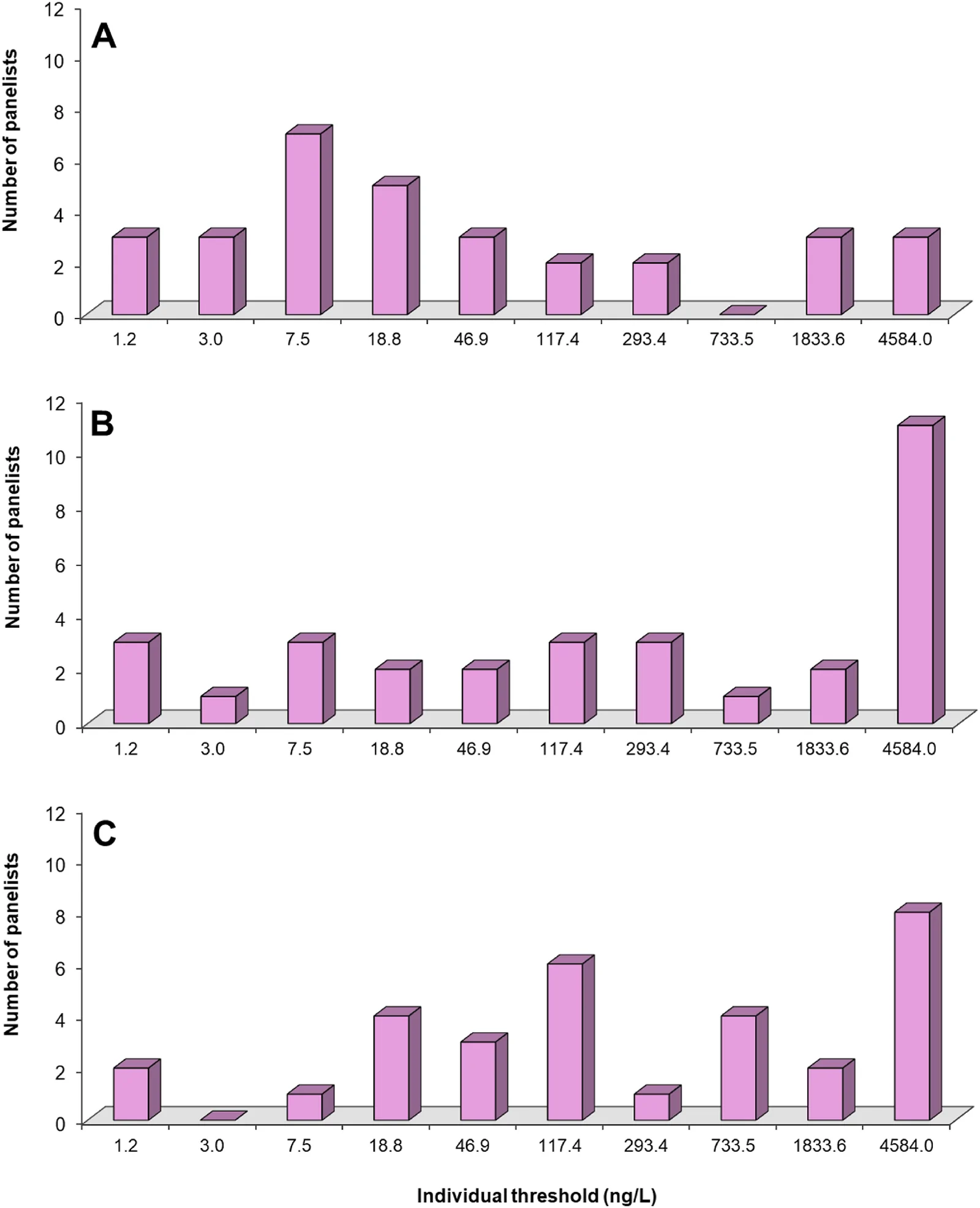
Distribution of individual thresholds for rotundone in simplified hydroalcoholic model wine at A) 0 % v/v, B) 6 % v/v, and C) 12 % v/v (n = 31).

At 0 % v/v ethanol, ODTs provide a baseline reflecting the olfactory sensitivity among individuals. For rotundone, the group ODT estimated as the geometric mean of individual ODT was 38.2 ×/÷ 13.3 ng/L, which is consistent with a previous study reporting a value of 55 ng/L obtained using a similar experimental procedure (*i.e.*, a 30 mL flask filled with 10 mL of rotundone solution), but applying a different test concentrations and data treatment method (*i.e*., ISO 13301). In that study, an individual threshold of 61.8 ng/L was used to segregate sensitive and hyposmic panelists, representing approximately a 50/50 distribution within the population, and suggesting an overall genetic basis for sensitivity to rotundone. Although the non-unimodal pattern of individual ODT distribution was confirmed statistically (*p* < 0.01, Hartigan's test), only 32 % of panelists exhibited an ODT ≥ 117.4 ng/L, and 19 % ≥ 1833.6 ng/L (Fig. 1A). Overall, although this does not fully contradict the hypothesis of a genetically based sensitivity, these results support the idea that age may also influence sensitivity to rotundone, as previously reported, with younger adults showing greater sensitivity (Geffroy et al., 2023).

**Fig. 2:**
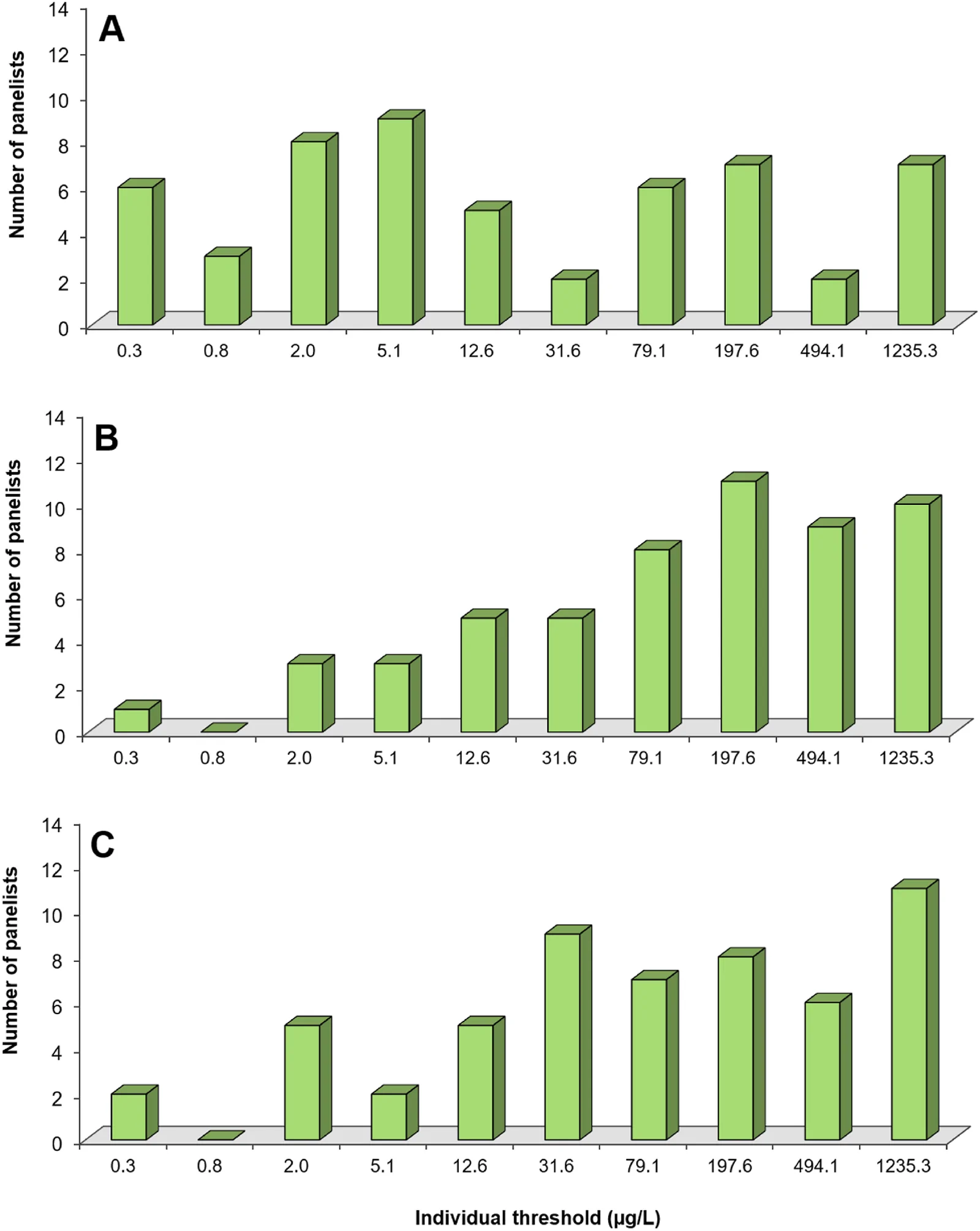
Distribution of individual thresholds for isoamyl acetate in simplified hydroalcoholic model wine at A) 0 % v/v, B) 6 % v/v, and C) 12 % v/v (n = 55).

For isoamyl acetate, the group ODT was 17.7 ×/÷ 14.2 μg/L, with values comparable to those reported in water in previous studies (*e.g.*, 19 μg/L in Sega et al. (1967)). Though a large range of variation in sensitivity has already been reported for this compound, the distribution in ODT for isoamyl acetate is known to follow a normal gaussian pattern (Menashe et al., 2007). To our knowledge, this is the first time that such a non-unimodal distribution (Fig. 2A), confirmed using Hartigan's test (*p* < 0.05), is observed. This could be the consequence of the very homogenous young adults population used in our study which may have contributed to minimize potential biases associated with age-related olfactory decline (Doty and Kamath, 2014). Although ODT values originate from discrete dilution steps, they were treated as continuous variables for the application of Hartigan's dip test. This approximation may affect the robustness of the test, and these findings, which require further validation, should be interpreted with caution.

**Fig. 3:**
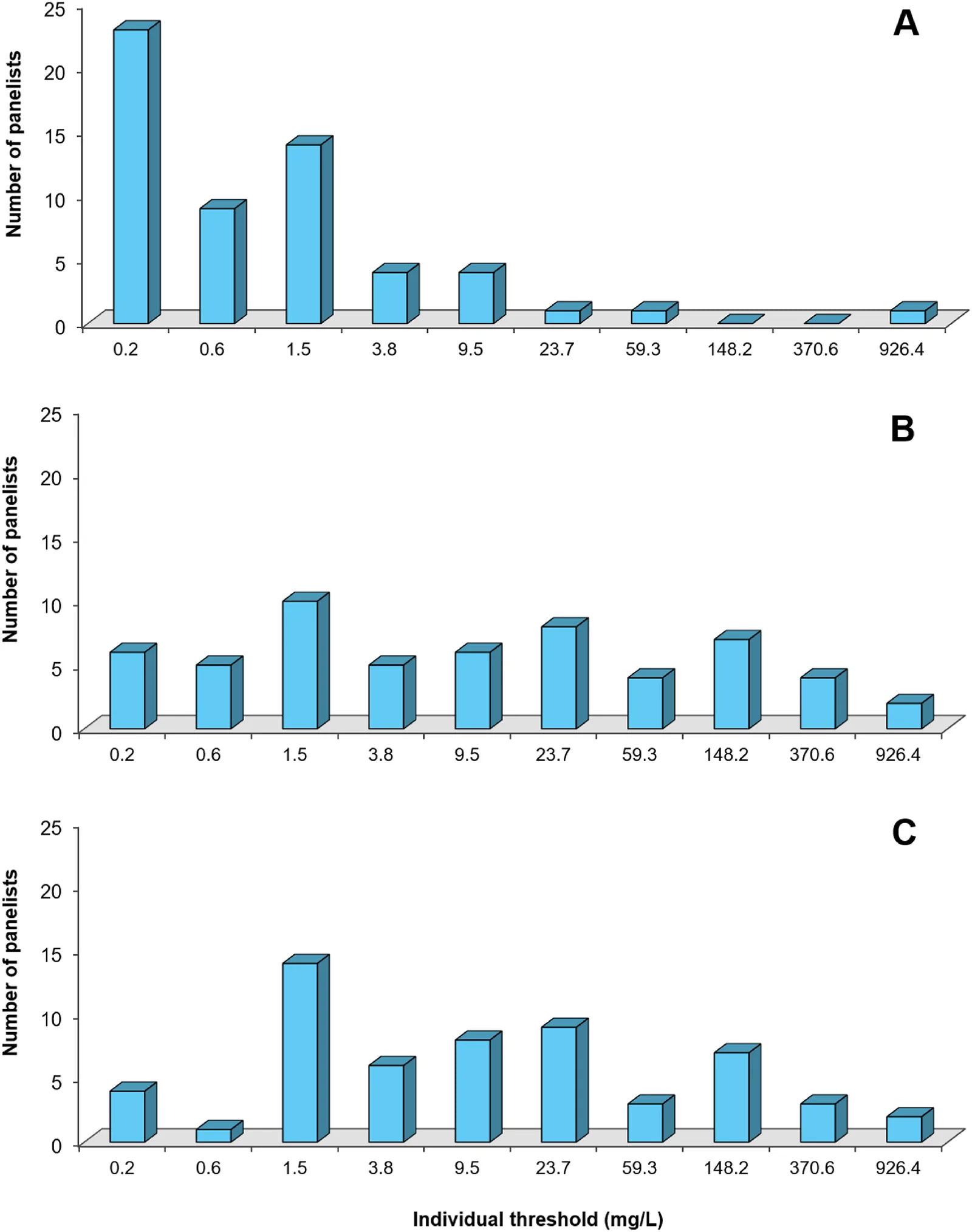
Distribution of individual thresholds for 2-phenylethanol in simplified hydroalcoholic model wine at A) 0 % v/v, B) 6 % v/v, and C) 12 % v/v (n = 57).

For 2-phenylethanol, the panel ODT was 0.95 ×/÷ 5.18 mg/L consistent with previous research on this aroma compound (Guth, 1997). The distribution of individual ODTs toward lower values, with 23 panelists successfully identifying the sample spiked with the lowest concentration of 2-phenylethanol, suggests that the ODT values obtained may have been overestimated and an additional series at 0.15 mg/L would have been valuable. However, given the strategy used in this study to maintain the same concentration across the three ethanol levels investigated, the distribution pattern observed at the two other ethanol concentrations (Fig. 3A and B) tends to support the relevance of our choice. Furthermore, such an addition would have been difficult to implement, as panelists already had to sniff a large number of samples during the session (*i.e*., 81).

### 3.2 Effect of ethanol on ODT for the three compounds

The effect of ethanol on the distribution of ODT can be observed in Fig. 1B and 1C for rotundone, 2B and 2C for isoamyl acetate, and 3B and 3C for 2-phenylethanol. Overall, the addition of ethanol shifted individual ODTs toward higher values, with a relatively even spread across the studied range for rotundone and 2-phenylethanol, whereas for isoamyl acetate, individual ODTs tended to shift toward higher values following a more Gaussian-like distribution.

**Fig. 4:**
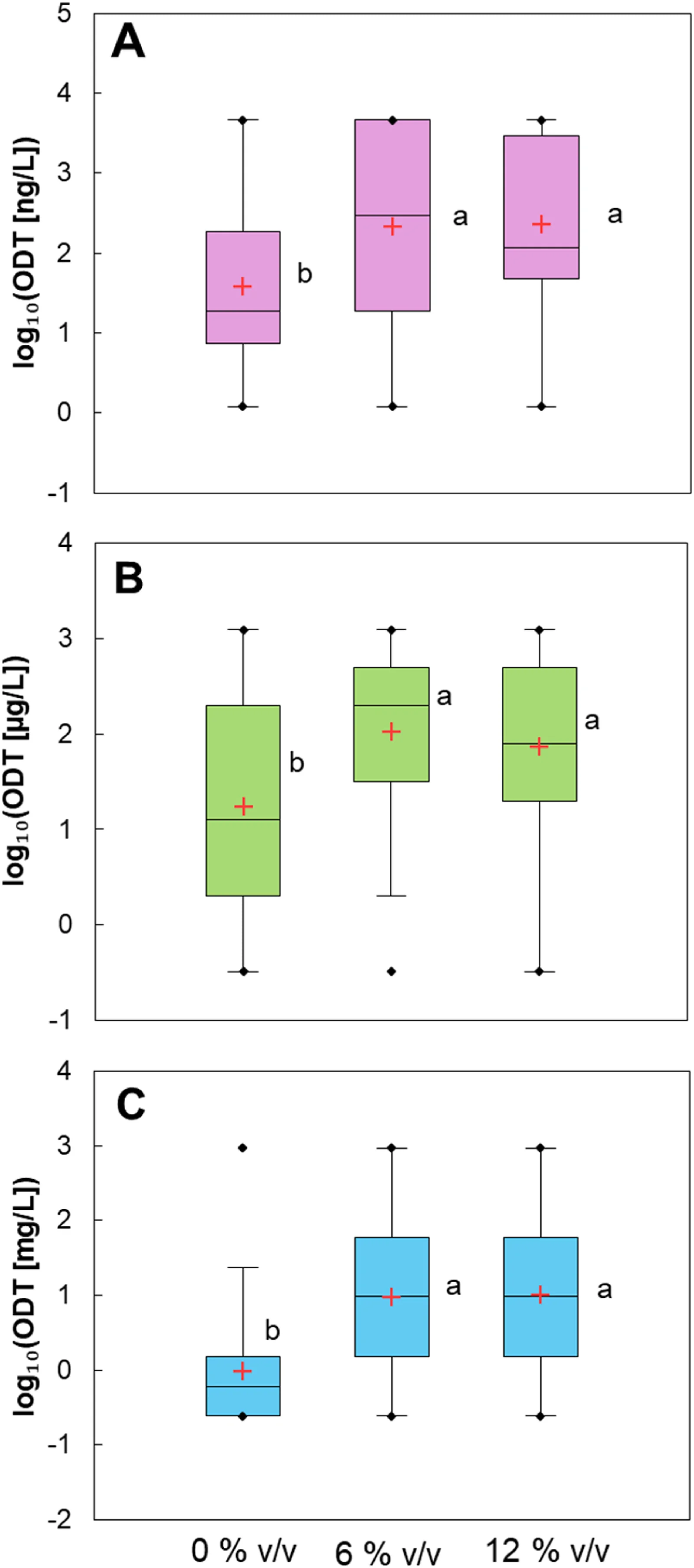
Impact of ethanol on log_10_ (ODT) in a simplified hydroalcoholic model wine for (A) rotundone (n = 31), (B) isoamyl acetate (n = 55), and (C) 2-phenylethanol (n = 57). ODT were expressed in ng/L, μg/L and mg/L, for rotundone, isoamyl acetate and 2-phenylethanol, respectively. Different letters for a given aroma compound indicate significant differences (*p* < 0.05) based on a linear mixed model (LMM).

For rotundone, ODTs were significantly higher at 6 % v/v (218.3 ×/÷ 19.5 ng/L) and 12 % v/v (231.6 ×/÷ 12.2 ng/L) compared to 0 % v/v (*p* < 0.01, LMM), corresponding to a 5.7 to 6.1-fold increase (Fig. 4A). For isoamyl acetate (Fig. 4B), a similar trend was observed (*p* < 0.001, LMM), with a 5.9-fold increase at 6 % v/v (104.9 ×/÷ 7.7 μg/L) and a 4.2-fold increase at 12 % v/v (74.0 ×/÷ 9.6 μg/L). Comparable significant results (*p* < 0.001, LMM) were obtained for 2-phenylethanol (Fig. 4C), with a 9.8-fold increase at 6 % v/v (9.34 ×/÷ 11.12 mg/L) and a 10.8-fold increase at 12 % v/v (10.28 ×/÷ 8.83 mg/L). For all compounds, no significant differences in ODT were observed between 6 and 12 % v/v. For isoamyl acetate, considering the number of panelists who failed to identify the correct sample at the highest concentration tested under the 6 and 12 % v/v conditions, it cannot be excluded that the group threshold was underestimated, which could have resulted in a higher fold-change compared to 0 % v/v. Consistent with this, the multiplicative dispersion around the 12 % v/v estimate (×/÷ 9.6) was wider than at 6 % v/v (×/÷ 7.7), pointing to greater uncertainty at the highest ethanol level and supporting an underestimation of the true threshold rather than a genuine reversal of the ethanol effect.

### 3.3 Effect of ethanol on generated sensory descriptors

The descriptor citation frequencies recorded per participant and subsession based on at least one citation across the nine series and the estimated headspace concentration through modeling are shown in Table 2, Table 3, respectively.

**Table 2:** Effect of ethanol on descriptor citation frequencies assessed by Chi-square test for the three aroma compounds studied. Descriptor occurrence was recorded per participant and subsession based on at least one citation across the nine series. Different letters for a line and a compound indicate significant differences by the Marascuilo procedure at *p* < 0.05.

Aroma compound	Descriptor	0 % v/v	6 % v/v	12 % v/v	*p*
Rotundone	Pepper-like	45.2 a	35.5 a	41.9 a	0.732
	Ethanol-like	16.1 a	32.3 a	29.0 a	0.307
Isoamyl acetate	Banana-like	38.2 a	32.7 a	27.3 a	0.476
	Ethanol-like	20.0 a	41.8 b	41.8 b	< 0.05
2-phenylethanol	Rose-like	52.6 a	52.6 a	59.6 a	0.685
	Ethanol-like	7.0 a	29.8 b	33.3 b	< 0.01

**Table 3:** Effect of ethanol on calculated headspace concentrations expressed in 10^−11^ mol/L at ODT concentrations for the three aroma compounds studied.

Aroma compound	Calculated headspace concentration at ODT (10^−11^ mol/L)		
	0 % v/v	6 % v/v	12 % v/v
Rotundone	0.0353	0.0174	0.0037
Isoamyl acetate	241	727	280
2-phenylethanol	7.38	48.2	35.2

For the three compounds, the citation frequencies of the expected aroma descriptors (*i.e.*, pepper-like for rotundone, banana-like for isoamyl acetate, and rose-like for 2-phenylethanol) did not differ significantly across the ethanol conditions (*p* > 0.05), suggesting that the aroma perception was preserved. In contrast, the frequency of ethanol-like descriptors increased significantly for isoamyl acetate (*p* < 0.05) and for 2-phenylethanol (*p* < 0.01) at 6 and 12 % v/v while no significant change was observed for rotundone.

However, these results should be interpreted with caution, as the expected descriptor may have been reported at concentrations well above the true ODT for each individual. In most cases, this descriptor was reported several times by a given panelist across the nine series, including near the determined ODT. Overall, these findings suggest that panelists clearly perceived the ethanol odor at higher concentrations, without substantially interfering with the recognition of the main aroma notes. Interestingly, the frequency of ethanol-like descriptors was not null for the 0 % v/v condition, even though added ethanol did not exceed 0.5 % v/v (§ 2.1). This could be attributed to the panelists’ limited training and relative inexperience in describing odors, as well as potential carry-over effects from previous series when the 0 % v/v samples were evaluated after the 6 or 12 % v/v conditions.

### 3.4 Effect of ethanol on volatility and headspace concentration modeling

**Fig. 5:**
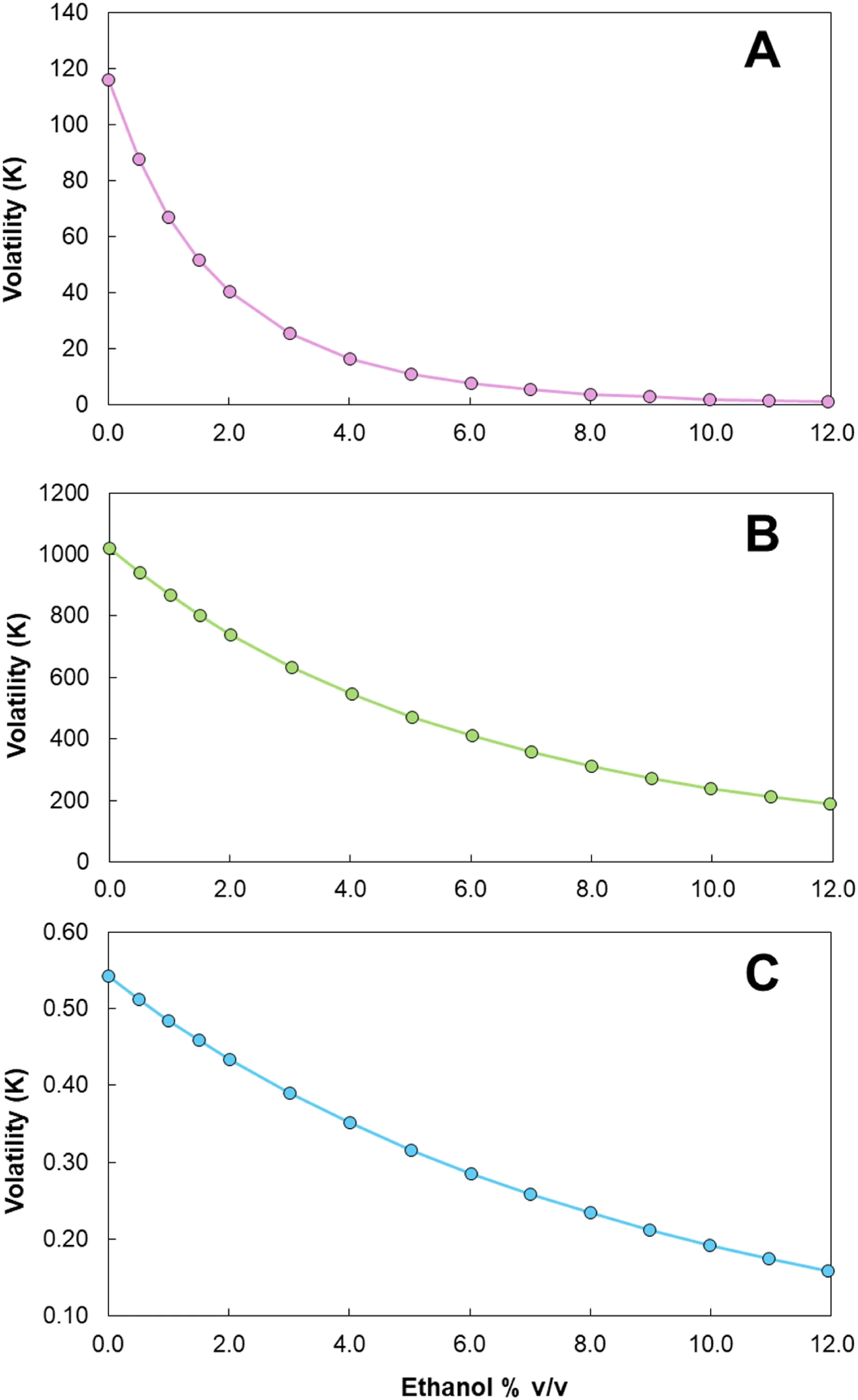
Evolution of computed volatility (K) at T = 20 °C in the aroma-water-ethanol ternary system as a function of ethanol for A) rotundone, B) isoamyl acetate, and C) 2-phenylethanol.

The effect of ethanol on volatility for the three compounds is illustrated in Fig. 5A, 5B, and 5C. Overall, isoamyl acetate showed the highest volatility among the three studied compounds, followed by rotundone and 2-phenylethanol. The markedly low volatility of 2-phenylethanol compared to the other two molecules and its tendency to be retained in the liquid phase may help explain its high detection threshold in the mg/L range. The calculated headspace concentrations at ODT provide interesting and complementary information (Table 3). Beyond molecule-receptor interactions, such values give an indication of the intrinsic olfactory potency of a given molecule. Although rotundone showed the lowest headspace concentration in mol/L at ODT, fewer molecules of 2-phenylethanol than of isoamyl acetate were sufficient to induce a stimulus, suggesting a higher olfactory potency for the former.

Overall, the decrease in volatility of aromas induced by ethanol was greater for rotundone (VEF_6→0_ = 14.9, VEF_12_ _→_ _0_ = 86.7), compared to isoamyl acetate (VEF_6→0_ = 2.5, VEF_12_ _→_ _0_ = 5.4) and 2-phenylethanol (VEF_6→0_ = 1.9, VEF_12_ _→_ _0_ = 3.4). These findings are consistent with previous observations emphasizing the highly hydrophobic character of rotundone (predicted LogP = 4.98) (Caputi et al., 2011).

## 4 Discussion

### 4.1 Relative contributions of thermodynamic and perceptual effects on ODT changes

**Fig. 6:**
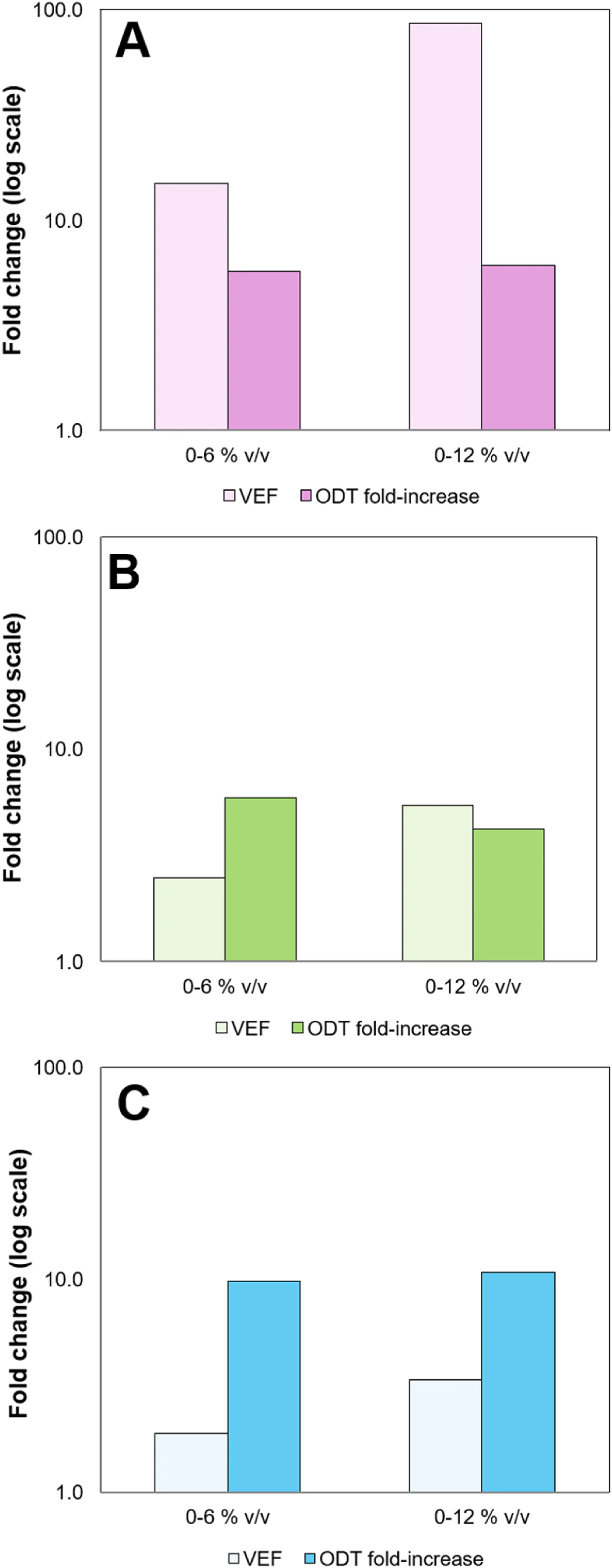
Comparison of thermodynamic (VEF) and sensory (ODT fold-change) effects of ethanol addition for (A) rotundone, (B) isoamyl acetate, and (C) 2-phenylethanol. VEF values represent the change upon ethanol decrease from 6 or 12 % (v/v) to 0 %, whereas ODT values represent the change upon ethanol increase from 0 to 6 or 12 % (v/v).

The combined calculation and sensory approach implemented in this study enables to assess the relative contributions of thermodynamic and perceptual effects by comparing the changes in volatility (as expressed by VEF) with experimentally observed changes in ODT (Fig. 6). For rotundone, VEF_6→0_ = 14.9 and VEF_12_ _→_ _0_ = 86.7 far exceeding the observed ODT fold-increases of 5.7 and 6.1, respectively. This discrepancy indicates that, despite the large thermodynamic reduction in volatility, fewer molecules, as illustrated by headspace concentration at ODT (Table 3), were sufficient to trigger detection. This pattern may be linked to the specific interaction of rotundone with the lipid bilayer of olfactory receptor cell membranes due to its high lipophilicity as proposed by Geffroy et al. (2018). For isoamyl acetate, while the observed ODT fold-increase at 12 % v/v (4.2) is broadly consistent with VEF_12_ _→_ _0_ = 5.4, the observed value at 6 % v/v (5.9) exceeds VEF_6→0_ (2.5), suggesting an additional perceptual contribution at this specific concentration as further supported by the higher headspace concentration at ODT (Table 3).

For 2-phenylethanol, VEF_6→0_ = 1.9 and VEF_12_ _→_ _0_ = 3.4, are well below the observed ODT fold-increases of 9.8 and 10.8, respectively, indicating that perceptual effects are the dominant driver of ODT elevation for this compound. This is further supported by the fact that VEF values were the lowest among the three compounds studied, yet the ODT fold-increases were the greatest.

The descriptor citation frequency data (Table 2) provide further support for this interpretation. The significant increase in ethanol-like descriptors for isoamyl acetate and 2-phenylethanol at 6 and 12 % v/v, while the expected aroma descriptors remained stable, is consistent with an ethanol odor masking effect in addition to the thermodynamic reduction in headspace concentration. In contrast, the absence of a significant change in ethanol-like descriptors for rotundone aligns with the predominantly thermodynamic nature of its ODT increase.

The more pronounced ODT elevation observed for 2-phenylethanol is therefore more likely attributable to a combination of thermodynamic effects and perceptual masking by ethanol. In addition, as the ODT at 0 % v/v for this aroma compound may have been slightly overestimated for the reason previously exposed, the actual differences between ethanol conditions might be even greater than those reported here, further supporting the predominance of perceptual effects.

The absence of significant differences in ODT between 6 and 12 % v/v provides additional insight into the underlying mechanisms. If thermodynamic effects alone were driving the increase in ODT, a more gradual and continuous rise would be expected with increasing ethanol concentration, reflecting the progressive reduction in aroma compound headspace concentration. Conversely, the observed stagnation suggests that the effect of ethanol may reach a limit within this concentration range, which is more consistent with a perceptual mechanism, where the ethanol masking effect may become saturated at relatively low concentrations. However, this perceptual masking effect within the 6 to 12 % v/v range has not been characterized as saturating in previous work on model wine (De-la-Fuente-Blanco et al., 2024).

Beyond thermodynamic and perceptual effects, other mechanisms may also contribute to the observed patterns. For rotundone specifically, the hypothesized partitioning into the lipid bilayer of olfactory receptor cell membranes due to its high lipophilicity (Geffroy et al., 2018) might play a role. Although a pharmacodynamic contribution is unlikely given the static conditions, it cannot be entirely ruled out, as ethanol has been shown to modulate olfactory receptor cell function in a compound-specific manner (Wang and Cadwallader, 2024).

### 4.2 Limitations, practical implications and further research

In the present study, glycerol, and organic acids were deliberately excluded from the simplified wine model to focus specifically on ethanol-aroma interactions and to maintain consistency between sensory measurements and thermodynamic modeling. The wine matrix is more complex, and non-volatile components such as glycerol, sugars, polysaccharides, and phenolic compounds can interact with aroma compounds, altering both their headspace partitioning and sensory perception (Villamor and Ross, 2013). Therefore, it cannot be excluded that distinct results would have been obtained in a more complex matrix although the trends observed across the different ethanol concentrations would likely have remained similar.

In the same way, the sensory panels for the three compounds consisted predominantly of young adults, mostly students. While this ensured a homogeneous population across the three sub-studies, it may not be representative of the broader wine-consuming population, and age- or experience-related differences in olfactory sensitivity should be considered when extrapolating these thresholds to other consumer groups.

It is possible that some sensory fatigue occurred, as most participants were not accustomed to the task and had to sniff a total of 81 samples during the session. However, the impact on the results is likely to be limited, since the order of the sessions (0, 6, and 12 % v/v) was fully randomized across participants. Although the same test solution concentrations were used across all ethanol levels to ensure consistency and comparability, it cannot be excluded that additional test concentrations would have been beneficial and would have provided a better ODT estimation especially for isoamyl acetate at 6 and 12 % v/v.

It can also be highlighted that blank samples were not solvent-matched for the small amount of ethanol contributed by the aroma stock solutions. However, the ethanol increase observed remained for all three compounds below 0.25 % v/v in most cases, with a maximum value of 0.42 % v/v corresponding to the highest aroma concentration tested, for which the ethanol effect is expected to be limited relative to the stimulus provided by the aroma compound. It is also well below the reported ODT of ethanol in water of 0.66 % v/v (Guttman et al., 2020), and is negligible relative to the base ethanol content of the 6 % and 12 % v/v matrices, where this potential bias is essentially eliminated by the much larger background level. For the 0 % v/v matrix, the measured ODTs for all three compounds closely matched literature values which makes it unlikely that panelists relied on an ethanol cue rather than the target aroma compound itself. Nevertheless, the use of solvent-matched blanks or other solvent such as dipropylene glycol as in a previous study (Geffroy et al., 2020) should be considered in future studies to further minimize any potential bias.

Despite substantial reductions in volatile concentrations due to the dealcoholization process, which can reach up to 90 % (Kumar et al., 2024), our results showed that ODTs at 0 % v/v were 4.2 to 10.8-fold lower than in the presence of ethanol, indicating that they may still occur at concentrations sufficient to have a substantial contribution to the overall aroma profile of fully, and particularly of partially dealcoholized wines where aroma losses may be less pronounced. This is particularly evident for 2-phenylethanol, the compound most strongly affected by ethanol. Regarding technologies such as SCC or GOLO that enable aroma recovery (Alises et al., 2025), our findings suggest that aroma compounds may contribute more markedly than in the native wine, although further investigation would be required.

To our knowledge, this study represents the first implementation of a combined perceptual and thermodynamic modeling approach for evaluating ethanol effects on ODTs in a static system. This modeling approach is particularly interesting to assess headspace gaseous concentrations because headspace sampling for trace GC analysis can be challenging, as it is known to perturb volatile equilibria in static conditions (Ettre, 2001). While static headspace studies are appropriate for controlled equilibrium measurements reflecting “first nose” conditions, the relevance of dynamic conditions for modelling aroma partitioning might remain limited. From a mass transfer and process engineering perspective, significant deviations from equilibrium would require continuous renewal of the gas phase, which is unlikely to occur during typical tasting practices.

## 5 Conclusions

The present study is the first to implement a combined thermodynamic modeling and sensory approach to assess the effect of ethanol on odor detection thresholds (ODTs) for three key wine aroma compounds (rotundone, isoamyl acetate, and 2-phenylethanol) in simplified hydroalcoholic model wine systems at concentrations relevant to the NoLo range. All three compounds showed significantly elevated ODTs at 6 and 12 % v/v ethanol compared to water, with no significant difference between the two ethanol levels. NRTL-based thermodynamic modeling revealed compound-specific enhancement in volatility at 0 % v/v that were greatest for rotundone, moderate for isoamyl acetate, and least pronounced for 2-phenylethanol. By comparing thermodynamically predicted volatility fold-increases (VEF_a→0_) with experimentally observed ODT fold-increases, the contributions of the two mechanisms could be distinguished. While the ODT increase for rotundone appeared predominantly thermodynamically driven, perceptual effects likely related to ethanol odor masking played a dominant role for 2-phenylethanol, with isoamyl acetate showing a mixed behavior. These findings have direct practical relevance for the production of dealcoholized wines, as the substantially lower ODTs measured at 0 % v/v ethanol suggest that the studied aroma compounds may still contribute meaningfully to the aroma profile of fully dealcoholized wines, even after the significant volatile losses associated with the dealcoholization process. More broadly, the combined approach and the volatility enhancement factor (VEF_a→0_) introduced provide a methodological framework readily transferable to other aroma compounds, hydroalcoholic matrices, and beverage categories where the role of ethanol on aroma perception remains poorly understood.

## CRediT authorship contribution statement

**Laura Brian:** Investigation, Visualization, Writing – original draft. **Anthony Santacreu:** Investigation. **Come de Tournadre:** Investigation. **Michel Malcor de Pierrefeu:** Investigation. **Jeanne Laronche:** Investigation. **Jean-Stéphane Condoret:** Supervision, Writing – review and editing. **Audrey Devatine:** Supervision, Writing – review and editing. **Séverine Camy:** Funding acquisition, Project administration, Supervision, Writing – review and editing. **Olivier Geffroy:** Conceptualization, Methodology, Formal analysis, Funding acquisition, Project administration, Supervision, Writing – original draft.

## Ethics declaration

Written informed consent to take part in the study and to publish the article has been obtained from all participants or their legal representatives. The privacy rights of participants have been observed.

This study was performed in compliance with relevant laws, regulatory frameworks and guidelines where the research took place. Ethics committee approval was not required under relevant laws and institutional guidelines. Ethical approval was not required for this type of sensory study at the École d’Ingénieurs de Purpan. However, all participants were informed about General Data Protection Regulation (GDPR) requirements and provided written consent.

## Data availability

Data will be made available on request.

## Declaration on use of generative AI in the writing process

The authors affirm that no generative artificial intelligence (AI) or AI-assisted technologies were used to create ideas, or scientific interpretations in the preparation of this manuscript. AI tools were employed solely to enhance language clarity and readability, under strict human oversight. All content was thoroughly reviewed and edited by the authors, who take full responsibility for the final version. The authors acknowledge that authorship entails responsibilities that can only be fulfilled by humans and have adhered to these principles throughout the manuscript preparation.

## Funding source

This project was supported by Toulouse INP and the École d’Ingénieurs de Purpan through funding of Laura Brian’s grant.

## Declaration of competing interest

The authors declare that they have no known competing financial interests or personal relationships that could have appeared to influence the work reported in this paper.
